# Review Of Internet Health Information Quality Initiatives

**DOI:** 10.2196/jmir.3.4.e28

**Published:** 2001-12-26

**Authors:** Ahmad Risk, Joan Dzenowagis

**Affiliations:** ^1^World Health Organization20 Avenue AppiaSwitzerland

**Keywords:** Internet/standards, Ethics, Professional, Social Control, Formal, Health Care Quality, Quality Assurance, Health Care/standards, Commerce/standards, Information Management/standards, Medical Informatics/standards, Quality control, Guidelines, Privacy, Informed Consent

## Abstract

**Background:**

The massive growth of health information on the Internet; the global nature of the Internet; the seismic shift taking place in the relationships of various actors in this arena, and the absence of real protection from harm for citizens who use the Internet for health purposes are seen to be real problems. One response to many of these problems has been the burgeoning output of codes of conduct by numerous organizations trying to address quality of health information.

**Objectives:**

Review the major self-regulatory initiatives in the English-speaking world to develop quality and ethical standards for health information on the Internet. Compare and analyze the approaches taken by the different initiatives. Clarify the issues around the development and enforcement of standards.

**Methods:**

Quality initiatives selected meet one or more of the following criteria: Self-regulatory. A reasonable constituency. Diversity (eg, of philosophy, approach and process)-to achieve balance and wide representation, and to illustrate and compare different approaches. Historic value. A wider reach than a national audience, except when its reach is a significant sector of the Internet health information industry.

The initiatives were compared in 3 ways: (1) Analysis and comparison of: key concepts, mechanism, or approach. Analysis of: the obligations that a provider has to meet to comply with the given initiative, the intended beneficiaries of that initiative, and the burdens imposed on different actors. These burdens are described in terms of their effect on the long-term sustainability and maintenance of the initiative by its developers. Analysis of the enforcement mechanisms. (2) Analysis and comparison by type of sponsoring organization, the reach of the initiative, and the sources of funding of the initiative or the sponsoring organization. (3) How the various initiatives fall under 1 of 3 key mechanisms and comparison of the advantages and disadvantages of these key mechanisms.

**Results:**

The issues that affect the initiatives and future work on the quality of health information on the Internet are identified and analyzed. These issues are:

(a) Three key mechanisms used in the quality initiatives (b) Sustainability issues that affect the initiatives: Burdens placed on health information providers, citizens and others. Currency and maintenance issues of the initiatives. Funding. Cost. Acceptance. Market conditions. User indifference or ambivalence. (c) Enforcement issues surrounding the initiatives (d) Adequacy of approach, scope, reach, and enforcement provisions of the various quality initiatives (e) Gaps that need to be addressed to achieve good quality of health information on the internet

**Conclusions:**

Ten conclusions are presented. A framework of action to be undertaken by the World Health Organization in the field of quality of health information on the Internet is recommended.

## Introduction

### Current situation of health information on the Internet

#### A global medium and a seismic shift

There is an explosion in the amount of health information available on the Internet. This increase does not show signs of slowing down. For example, entering the word "health" in a generic search engine like Google (www.google.com) currently yields over 60 million pages.

The sources of that information are numerous and varied. For the first time in history, we have a global medium that transcends geography and operates across cultures and languages.

The Internet has been the catalyst for the seismic shift that is happening in the doctor-patient relationship. It continues to have a profound impact on other relationships among health care actors. This shift has been in access to knowledge, and consequently in access to power [1,2].

Numbers vary and none are very accurate, but it is estimated that there are over 100,000 health-related Web sites on the Internet today. These vary from highly-academic sites, online peer-reviewed journals, governmental sites, and health-provider-institutions' sites to countless individual contributions from citizens, patients, and health professionals.

There is also an unmeasured number of industry-related Web sites, ranging from large and small pharmaceutical company sites to a multitude of commercial sites disseminating information or selling products and services in a variety of bewildering ways.

Recent surveys estimate the number of Internet health-information seekers to be about 86% of the estimated 168 million American adults who have access to the Internet [3], and that 55% (Germany) to 90% (United States) of primary-care physicians had ever used the Internet (P/S/L Research [4] i-MD 2000 Survey. 2nd Quarter 2000; currently, survey is not available at the P/S/L Research Web site). These surveys indicate that the trend towards use of the Internet for health purposes is rising.

In addition, we are beginning to witness the large-scale entry of mainstream health care organizations into the field of the health Internet. There are an increasing number of purchaser organizations and health-care-funding governments that are exploring the use of the Internet as a tool for containing the spiraling cost of health care and improving the quality of care to citizens.

Numerous surveys and studies paint a picture of dubious information quality, widespread practice of fraud, potentially-dangerous claims, and the risk of exposure of citizens to harm. One good example of such surveys is the study conducted by RAND health [5].

Even when information appears to be of high quality it can cause unintentional harm to citizens [6]. This can happen for a number of reasons:

- Language and complexity barriers [7]
- Inappropriate audience or context
- Unavailability of certain services or products in different parts of the world
- Difficulty in interpreting scientific data
- Accuracy and currency of information
- Potential for source bias, source distortion, and self-serving information

#### No real protection

Amid all this disorder, there is a common concern among many individuals and institutions interested in the health Internet. This concern is for the prevention of physical, mental, and emotional harm-caused by wrong, misleading, inappropriate, false, fraudulent, or self-serving information-to people who use the Internet to seek or receive health information, products, and services.

Yet, in a large number of Web sites currently offering health information we cannot find credible and enforceable protection of citizens from potential harm.

While there is some degree of protection provided either by national regulatory mechanisms or through self-regulation, this modest protection is currently only afforded to a small number of people.

#### The response

One response to many of these problems has been the burgeoning output of codes of conduct from numerous organizations trying to address quality of health information. All of these codes have a primary goal of citizen protection, and some have a secondary goal of protecting the company's "good name," thus succeeding in competition based on quality. These initiatives derive from different philosophies and apply different approaches and processes.

#### This paper

This paper reviews and compares major self-regulatory initiatives for health information quality and ethics developed in the English-speaking world.

### Scope

This paper analyzes the major quality initiatives for health information on the Internet. The criteria for inclusion in the review are discussed in "Methods, " below.

The focus of this study is health information, while being mindful of the inevitable overlaps between information and products and services. This study adopts the definition of health information of the eHealth code of Ethics in view of its accuracy and completeness. The eHealth Code of Ethics defines health information as:

Health information includes information for staying well, preventing and managing disease, and making other decisions related to health and health care. It includes information for making decisions about health products and health services. It may be in the form of data, text, audio, and/or video. It may involve enhancements through programming and interactivity.

This paper does not address:

- Provision of organized health care services
- The practice of telemedicine
- Laws and regulatory instruments
- Quality initiatives developed or being developed by non-English speaking groups or organizations

The review does not perform a competitive analysis of the various initiatives.

This paper avoids the use of the neologism *eHealth* because of its ambiguity. Rather, it uses the terms *health Internet, health Internet information*, or *health information* referring broadly to the use of information and communication technologies to create, deliver, or receive health information with particular reference to Internet technologies.

### Objectives

Provide a comprehensive review of the key efforts to develop quality and ethical standards for health information on the Internet.

Provide comparison and analysis of the approaches taken by the different initiatives.

Clarify the issues around the development and enforcement of standards for health information on the Internet.

## Methods

The selection of quality initiatives for review and comparison in this paper is based on meeting one or more of the following criteria:

- The initiative is an expression of a self-regulatory mechanism. This study focuses on those initiatives that aim to provide models of self-regulation of the health Internet industry. Legal and regulatory mechanisms are the subject of a separate paper. Self-regulation of the health Internet remains a powerful driver of the pursuit of quality standards for health information on the Internet.
- The initiative has a reasonable constituency, that is, a body of developers and followers who are keen on sustaining and maintaining the initiative-or the initiative has been developed by a broad spectrum of people. Issues of sustainability and maintenance are important components of the comparisons that follow.
- Diversity of philosophy, approach and process, and other characteristics-to achieve balance and wide representation, and to illustrate and compare different approaches.
- The initiative has some historic value representing an early example of thinking on quality standards. This criterion resulted in the inclusion of two early examples from 1996 whose current utilization is unknown.
- The initiative has a wider reach than a national audience, except when its reach is a significant sector of the Internet health information industry, for example, pharmaceutical Web sites or large commercial consortia. Many initiatives did not reach beyond national constituencies. In some cases, the geographical reach is not easily defined or crosses 2 different boundaries. For example, the MedCERTAIN project is classified in this paper as having a regional reach (in view of its European base and funding) but it also has ambitions to develop the project into an international standard. Another example is the code of conduct of the American Medical Association (AMA). Although it is intended to cover those sites under the control of the AMA, it also states that it can be utilized by any medical website.

The process of identifying the initiatives reviewed was based on personal knowledge of, involvement with, and exposure to the field of quality-of-health-information on the Internet.

The initiatives were compared in 3 ways:

1. Analysis and comparison of the different key concepts, mechanisms, or approaches. The analysis also looks at the obligations that a provider has to meet in order to comply with the given initiative, the intended beneficiaries of that initiative, and the burdens imposed on different actors. These burdens are described in terms of their effect on the long-term sustainability and maintenance of the initiative by its developers. Finally, the enforcement mechanisms applicable to the initiatives are looked at. (Table 1)
2. Analysis and comparison by type of sponsoring organization, the reach of the initiative, and the sources of funding of the initiative or the sponsoring organization. (Table 2)
3. Finally, how the various initiatives fall under one of 3 key mechanisms are looked at (Table 3). The advantages and the disadvantages of these key mechanisms are compared in the "Discussion" section. Briefly, these key modes are:
  - Codes of conduct
  - Third-party certification
  - Tool-based evaluation (for example, questionnaires that are filled by hand or embedded software that automatically gives access to the quality attributes of the site)

### Review of the initiatives

Much of the information about the initiatives has come from the published initiative; discussions with some of the key players in each initiative, and attendance at conferences on quality of health information on the Internet

For each initiative, the review is divided into the following areas (however, review of some initiatives did not involve discussion of all the areas):

- **Launch Date**
- **Responsible Organization**
- **Key players**
- **Intended target users**
- **Objectives**
- **Approach**
- **Process**
- **Implementation mechanisms**
- **Sustainability issues**

The actual text of the initiatives was used to describe the various aspects of the work on many occasions. On other occasions, the author provided descriptions and interpretations based on his own sources of information and his current understanding

**Table 1:** Characterization of quality initiatives

Initiative	Philosophy	Mechanism	Implementation Obligations	Intended Beneficiary	Sustainability	Burden Bearer	Enforcement
eHealth Code of Ethics	Code of conduct	Guidance	Interpret and specify guiding principles	Citizens	Currency Funding	Providers Citizens	None
HI-Ethics	Third-party certification or Voluntary compliance with code of conduct	Quality seal	Comply with code of conduct	Consumers Member companies	Currency Market Hi-E Inc	Providers Citizens	Withdrawal of accreditation Withdrawal of membership
URAC	Third-party certification	Accreditation process	Comply with accreditation process	Companies	Cost Acceptance	Providers Citizens	Withdrawal of accreditation
MedCERTAIN	Voluntary meta tags Trust mark Third-party certification	Meta tagging by provider Citizen assesses based on tags or rating or Sees trust mark	Comply with vocabulary Apply tags Third-party certifiers use tags	Citizens	Currency Acceptance Human resources Funding	Providers Raters Citizens	None Withdrawal of accreditation (when site is rated by third party)
TNO QMIC	Third-party certification	Accreditation process	Comply with accreditation process	Companies	Cost Acceptance Other Trusted Independent Parties (TIPs)	Providers Citizens	Withdrawal of accreditation
HON	Code of conduct	Quality seal	Comply with code of conduct	Citizens	Currency Market Human resources Funding	Providers Citizens	None
EC Quality Criteria	Quality criteria	Guidance	Comply with criteria	EU (European Union) member states	Relevance Interpretation Political commitment	Providers Member states Citizens	None
OMNI	Third-party evaluation based on quality criteria	Manual filtering	Comply with quality criteria	Academe	Currency Human resources Funding Raters	Providers Citizens	None
DISCERN	Tool-based assessment	Tool-based filtering	Comply with quality criteria	Citizens	Currency	Providers Citizens	None
AMA	Code of conduct	Self-regulation of own sites	Comply with code of conduct	AMA	Currency	Providers	Enforced by AMA
BHIA	Code of conduct	Guidance	Comply with code	Citizens	Currency	Providers Citizens	None
HSWG IQ Tool	Tool-based evaluation	Tool-based rating	Comply with quality criteria	Citizens	Currency Funding	Providers Citizens	None
IFPMA	Code of conduct	Guidance	Comply with code	Member companies	Currency Specificity	Providers Citizens	None

This review looks at these initiatives:

1. eHealth Code of Ethics
2. Health Internet Ethics (Hi-Ethics)
3. URAC Health Web Site Accreditation Program
4. MedPICS Certification and Rating of Trustworthy and Assessed Health Information on the Net (MedCERTAIN)
5. TNO Quality Medical Information and Communication (QMIC)
6. HON Code
7. EC (European Community) Quality Criteria for Health-related Websites
8. Organizing Medical Networked Information (OMNI)
9. DISCERN
10. American Medical Association (AMA): Guidelines for Medical and Health Information Sites on the Internet: Principles Governing AMA Web Sites
11. British Healthcare Internet Association (BHIA): Quality Standards for Medical Publishing on the Web
12. The Health Summit Working Group-Criteria for Assessing the Quality of Health Information on the Internet: IQ Tool (HSWG IQ Tool)
13. The International Federation of Pharmaceutical Manufacturers Associations (IFPMA) Code of Marketing

**Table 2:** Quality initiatives: Sponsors, Scope, and Funding

Name of initiative	Type of Organization		Reach			Funding			
	Voluntary	Commercial/ Corporate	National	Regional	International	Donations	Members	Fee for Service	Public Money
eHealth Code of Ethics	**•**				**•**	**•**	**•**		
Hi-Ethics	**•**		**•**				**•**		
URAC		**•**		**•**				**•**	
MedCERTAIN	**•**			**•**					**•**
TNO QMIC		**•**	**•**					**•**	**•**
HON Code	**•**				**•**	**•**			**•**
EC Quality Criteria	**•**			**•**					**•**
OMNI	**•**		**•**						**•**
DISCERN	**•**		**•**						**•**
AMA		**•**	**•**				**•**		
BHIA	**•**				**•**		**•**		
HSWG IQ	**•**	**•**			**•**				
IFPMA	**•**				**•**		**•**		

**Table 3:** Quality Initiatives: Key Mechanisms

Codes of Conduct/Ethics	Third-party Certification or Rating	Tool-based
1. eHealth Code of Ethics 2. Hi-Ethics 3. HON Code 4. EC quality criteria 5. AMA 6. BHIA 7. IFPMA	1. URAC 2. MedCERTAIN 3. TNO QMIC 4. OMNI	1. DISCERN 2. IQ Tool

### eHealth Code of Ethics [8-9]

**Launch date**

24 May 2000.

**Responsible organization**

The Internet Healthcare Coalition.

Key concept: the Code sets out ethical concepts that inform the processes of self-assessment and compliance based on interpretation and specification.

The Internet Healthcare Coalition is a not-for-profit organization whose mission is to enhance quality health care resources on the Internet. It aims to achieve its mission by consumer and provider education, self-regulation, and the nurturing of on-line communities that promote ethical, innovative, and high-quality sources of health care information and services.

Membership of the Coalition comprises publishers of professional and consumer health care information; academic institutions and other accredited educational providers; medical libraries and database providers; medical specialty and special-interest societies; patient advocacy and support groups; manufacturers of regulated drugs and medical devices; and commercial developers and providers of Internet-based health-related education, information, and services.

The Coalition is funded by membership fees, unrestricted educational grants and donations, and proceeds from conferences and educational activities.

**Intended target users**

The eHealth Code of Ethics is developed as a set of guiding principles aimed at health Internet stakeholders worldwide. These stakeholders include health-application developers; site sponsors; managers; Webmasters; clinicians; laypeople who seek health information, products or services via the Internet; policy makers; academics; and publishers.

**Objectives**

- Protect from harm
- Create ethical environment
- Ensure fairness and synergy amongst the various entities

The goal of the eHealth Code of Ethics is to ensure that "people worldwide can confidently and with full understanding of known risks realize the potential of the Internet in managing their own health and the health of those in their care."

Thus, the Code has the overarching goal of identifying the values that are important in creating conditions of trust. The Code defines the kinds of conduct that support those values in practice. This becomes the foundation for enabling people to use the health Internet with confidence.

**Approach**

The approach is geared towards producing a set of overarching ethical principles for the health Internet that can provide guidance to further interpretation, specification, and development of ethical codes of conduct.

**Process**

- Grass root participation in development
- Democratic broad stakeholder consensus
- Professional-ethicists input
- Prior identification of the issues of concern through an on-line questionnaire undertaken by the Internet Healthcare Coalition
- Supply-side and public education
- Preparation and collection of case studies and interpretative guidelines

**Implementation mechanisms**

The Code together with its case studies and interpretative materials components is used in a number of ways:

- As the basis for a number of operational implementation activities, for example, those being developed by URAC, Kaiser Permanente, and the USA National Mental Health Association.
- As the basis for the series of eHealth Ethics workshops organized by the Coalition to inform and educate organizations that provide health information on the Internet on the issues of ethics and quality.
- The process deployed in developing the eHealth Code of Ethics is being used in developing other initiatives. Examples include the MedCERTAIN project and the European Commission's workshop on quality criteria for health-related Web sites
- The cochairs of the Summit and its Steering group, in common with the key players of the other initiatives reviewed here, play key roles in dissemination of the guiding principles of the Code, encouraging the adoption and adaptation of the Code and facilitating the development of standards in many international arenas.

**Sustainability issues:**

The eHealth Code of Ethics places a burden on other organizations developing quality standards for Internet health information in terms of those organizations having to (a) interpret and specify the Code according to the constituency addressed, as these activities will have to be supported by commitment of time and resources; and (b) be in compliance with the Code in broad terms.

Sustainability of the Code itself and its further development is vulnerable to scarcity of resources and commitment.

The burden of codes of conduct in general is ultimately passed onto the citizen. In the absence of real enforcement citizens are required to be interested, knowledgeable, and caring, with the desire and commitment to apply critical appraisal of sites proclaiming to be in compliance of a particular code.

### Health Internet Ethics (Hi-Ethics) [10]

**Launch date**

7 May 2000.

**Responsible organization**

Hi-Ethics Inc [11].

Key concept: third-party certification.

Hi-Ethics Inc is a not-for-profit consortium of US-based commercial health Internet companies. Current membership is 15 companies. Member companies provide the funding for the initiative through membership fees. Current membership fees are $6000.

**Intended target users**

US-based commercial Web sites: that offer or plan to offer health services, products, and information to consumers; that comply with the Hi-Ethics Principles; and that pay the applicable membership fee.

**Objectives**

The rules developed by Hi-Ethics are intended to assure that:

- Internet health services reflect high quality and ethical standards
- Health information is trustworthy and up-to-date
- Personal information is protected
- Consumers are able to distinguish on-line health services that follow the Hi-Ethics principles from those that do not
- Member companies' good names are maintained
- Self-regulation remains the primary mode of oversight
- Conformance with the applied principles serves as a means of legal defense and for verification procedures

**Approach**

The founding members of Hi-Ethics were motivated to approach the issue of health Internet ethics following media and consumer criticisms of commercial Web site practices, particularly in the areas of trust, privacy, and confidentiality. Other areas of concern that triggered the Hi-Ethics initiative were editorial integrity and advertising policies. Some argue that the founder of drkoop.com called for the initiative following specific criticism of his company.

This approach required: emphasis on high ethical standards, gaining the trust and confidence of the consumer, self-regulation, a framework for legal defense, and the establishment of a set of clear rules of conduct that sometimes go into great operational detail.

Hi-Ethics Inc also sees the need for governmental policy setting as well as the corporation's engagement in lobbying and educational activities.

**Process**

Establishing the Hi-Ethics Principles required the cooperation and collaboration of large commercial Web companies that were often in direct competition with one another, and whose business models and technology infrastructure were often very different from one another.

To ensure a level playing field and to address the issues of competition and the different business models of its members, Hi-Ethics Inc chose to have all decision-making processes require unanimous agreement. The law firm of Hogan and Hartson was retained to ensure that the Hi Ethics Principles could stand up to the rigor of any verification requirements either by regulatory authorities or through third-party certification.

It can be argued that the founding members of Hi-Ethics have acted with enlightened self-interest to obtain unanimous consent of the consortium in order to achieve the goal of setting the governing principles for the intended users.

**Implementation mechanisms**

Direct implementation by health Internet companies who meet membership criteria, and undergo a third-party certification process through the cooperative program between Hi-Ethics Inc and URAC for website accreditation.

The number of companies who have implemented the Hi-Ethics principles fully is not known.

**Sustainability issues**

Sustainability of the Hi-Ethics code of conduct is vulnerable to the burdens placed on citizens, member companies, prevailing market conditions, and the ability of Hi-Ethics Inc to maintain the currency of the principles.

This will be explored further in the "Discussion" section.

### MedCERTAIN [12- 13]

MedPICS [14] Certification and Rating of Trustworthy and Assessed Health Information on the Net

MedPICS is now replaced with HIDDEL (Health Information Disclosure, Description and Evaluation Language) [15]

**Launch date**

2000.

**Responsible organization**

This is an EU (European Union)-funded demonstration project under the "Action Plan on Promoting Safer Use of the Internet by Combating Illegal and Harmful Content on Global Networks" [16].

MedCERTAIN is a system based on metadata tagging technology, standard quality vocabulary, and content filtering labels. It relies on the cooperation of individuals and organizations that evaluate, assess, accredit, or recommend health information on the Internet to apply these technologies to their production processes.

The project is managed by a Project Consortium, which comprises 3 core partners:

- The University of Heidelberg, Department of Clinical Social Medicine
- The University of Bristol, Institute for Learning and Research Technology at the University of Bristol (ILRT)
- Finnish National Research and Development Centre for Welfare and Health (STAKES) / The Finnish Office for Health Care Technology Assessment (FinOHTA)

In addition, the project draws on the resources of the "Heidelberg Collaboration,", a loose collaboration based on the "Collaboration for Critical Appraisal of Internet Health Information," proposed in 1997.

**Intended target users**

Information providers and rating organizations (which include every organization, portal, or subject gateway active in recommending, evaluating, or endorsing health information or health-information providers), and ultimately, the end user of health information.

**Objectives**

- Establish self and third-party rating systems that enable consumers to filter harmful health information and to identify and select high-quality information ("downstream filtering") through Web site content labels
- Creation of an enforcement infrastructure
- Consumer education
- Active encouragement of information providers to conform to ethical codes of conduct
- Information providers and rating facilities achieve this through the application of meta tags and labeling technologies

**Approach**

- Metadata self-labeling by information providers
- Third-party rating and the award of a trust mark
- Standard metadata vocabulary, which draws on other quality initiatives like the eHealth Code of Ethics and the DISCERN questionnaire

**Process**

- Standard European Commission project management routines
- Input from the Heidelberg Collaboration
- Feedback from medical Webmasters on the MedPICS draft metadata vocabulary and rating criteria

**Implementation mechanisms**

- Information providers describe their content using the standard quality vocabulary and meta data technologies, for example, XML (Extensible Markup Language )
- These descriptors would act as labels that allow users to filter content according to personal criteria
- The same labels would also feed through to labeling bureaus by third-party rating facilities (for example, URAC), search engines, and health Internet Web sites
- Trust mark: MedCERTAIN defines 4 levels for the award of a trust mark:
  Level I: Transparency Mark (self-certification)
  Level II: Verification of Level I claims and formal assessment of the Web site by professional volunteers based on the quality criteria
  Level III: Third-party assessment and rating of content
  Level IV: Outcome evaluation

**Sustainability issues**

Sustainability of MedCERTAIN is dependent on certain conditions that have to be met. These are:

- Sufficient acceptance and implementation of meta tags by providers
- Correct interpretation and specification of the extensive quality vocabulary by information providers
- The emergence of strong third-party description and annotating organizations
- Progress in browser technology (to allow user-specified quality preferences) and wide acceptance of XML and meta tags standards
- Citizens being aware of the quality labels, having an interest in using them, and being able to interpret them

MedCERTAIN places burdens on citizens, providers, and third-party certification bodies.

**Update**

MedCIRCLE (Collaboration for Internet Rating, Certification, Labeling and Evaluation of Health Information) will use HIDDEL to describe other Web sites as "inner circle" and a loose collaboration of other subcontractors or non-funded partners as "outer circle," all using HIDDEL. MedCIRCLE is a collaboration of 3 national gateways in: Germany (Ärztliche Zentralstelle Qualitätssicherung-German Medical Association), Spain (Medical College of Barcelona), and France (CISMeF) [17].

DAERI [18] (Database of Adverse Events Related to the Internet) project, which is not directly part of MedCERTAIN, is somewhat related to the subject of this study in terms of providing useful feedback to quality processes. This is achieved through the collection of case studies of situations where patients have been harmed by information on the Internet.

### URAC Health Web Site Accreditation Programme [19- 20]

**Launch date**

August 2001.

**Responsible organization**

URAC (formerly known as the American Accreditation Healthcare Commission) [21].

URAC is a not-for-profit organization founded in 1990 to establish standards for the managed care industry. URAC's broad-based membership includes representation from all the constituencies affected by managed care: employers, consumers, regulators, health care providers, and the workers' compensation and managed care industries.

Member organizations of URAC participate in the development of standards, and are eligible to sit on the Board of Directors. URAC offers 10 different accreditation programs for managed care organizations.

More recently, URAC embarked on developing a program for the accreditation of health-related Web sites. The formulation of the program is now completed and it has been approved by the board of directors of URAC. It has undergone beta testing by selected health Internet organizations and is fully operational as of August 2001.

URAC primarily derives its funding from fees paid by applicants for accreditation,

**Intended target users**

Health-related Web sites, initially those organizations providing managed care services.

**Objectives**

- Address the concerns of consumers and other health care stakeholders
- Provide a tool to identify Web sites that meet high standards for quality and accountability

**Approach**

URAC approached the development for this accreditation program in the same way it approaches other non-Internet accreditation programs.

This approach involved appointing an advisory committee composed of expert representatives of all stakeholders. This committee follows standard URAC procedures in its work.

The approach relies on adaptation of existing quality initiatives; interpretation and specification for the target constituency; consensus; and public consultation and drafting, until a fully-operational program can be presented to the URAC Board of Directors for approval.

**Process**

URAC brings together experts in the field to debate and discuss what standards are appropriate for a particular aspect of health Internet information. The standards-development process is inclusive and broad-based-URAC membership itself includes a balance of organizations representing providers, regulators, businesses, consumers, and the health Internet information industry.

**Implementation mechanisms**

Health Web site organizations that wish to seek accreditation from URAC submit documentation of compliance with each standard. A member of URAC accreditation staff reviews this document, working closely with the applicant to resolve any issues that have been identified. URAC staff visits the applicant to ensure that its operations are consistent with the documentation submitted. Finally, the Accreditation Committee and the Executive Committee review the application. These committees are composed of representatives of URAC's member organizations.

An important requirement of the accreditation program is that the applicant demonstrates that it has established an organizational quality committee to oversee the ethical Internet operations of the organization.

URAC has set preliminary accreditation fees of $2000 to $5000, plus travel fees for URAC certifiers, for on-site inspections.

**Sustainability issues**

The success of the URAC program depends on:

- Sufficient acceptance by fee-paying customers to make the program viable
- Favorable market conditions in the health care industry in general and the health Internet sector in particular
- The ability of URAC to maintain the currency of its program of accreditation
- The value, if any, attached to accreditation by citizens

URAC places burdens on citizens through the need to understand and assess the quality criteria applied to the sites and the accreditation process behind it, and on its customers through financial and organizational burdens.

### TNO Quality Medical Information and Communication (QMIC)-Quality for medical information communication and transactions

**Launch date**

January 2001.

**Responsible organization**

Health Trust, part of the Netherlands TNO Prevention and Health Institute [22].

TNO (Applied Scientific Research) Institutes are independent organizations that were set up by the Netherlands government to act as bridges between science and society. The institutes are partly funded by public funds and partly by fees for services.

QMIC aspires to be the Netherlands Trusted Independent Party (TIP), while it hopes to become an "international facilitator for domain specific trusted independent parties." The scope and functions of the latter role lack clarity.

This will be a fee-for-service system; such fees will be in line with conventional ISO (International Organization for Standardization) accreditation fees. TNO sees the need for accreditation aggregators that could offer services at much-reduced rates to small enterprises.

**Intended target user**

Trusted Independent Third Parties (TIPs) whether existing or yet to emerge.

**Objectives**

Perform a capability assessment of the information suppliers on their ability to verify conformity with the requirements ("self-certification with external reference").

**Approach**

The TNO approach is third-party certification.

The core team of TNO QMIC comprised 3 individuals whose backgrounds are from the certification and accreditation industries.

This core team was later expanded to 10 people to include IT specialists and other conformity, standards, and process-flow specialists. The team is advised by an unknown number of physicians and informatics specialists.

**Process**

Classic International Organization for Standardization (ISO) process routines based on consensus, industry-wide solution, and voluntary compliance [23].

The process involves 6 stages:

- Proposal
- Preparatory
- Committee
- Enquiry
- Approval
- Publication

**Implementation mechanisms**

QMIC is an instrument based on the ISO 9000 and ISO 2000 accreditation procedures and has its roots in the certification and accreditation culture within the framework of the European New Approach Directives.

The system relies on 2 types of bodies: an intra-organizational Notified Body Function (NBF) (a compliance committee) and an external Trusted Independent Third Party (TIP) that performs the audits and accreditation.

TNO QMIC accreditation involves the following procedures:

Initial audit of the organization applying for accreditation conducted by the TIP:

- Capability assessment (information-domain specific). The capability assessment is a dynamic and incremental process that determines the level of authorization that the NBF would receive from the TIP
- Risk assessment of the impact of the health information under examination
- Regulatory compliance if applicable

The Notified Body Function is an independent intra-organizational body that deals with quality functions. The main functions of the NBF are the scrutiny of documents produced by the organization, confirmation of compliance with standards set, and ultimate release of documents for publishing onto the Web site. The TIP supervises all activities of the NBF.

The organization would "'notify" the TIP, which in turns carries out the capability assessment and issues the necessary "certification.". Additionally, the NBF issues the organization with self-certificates for the day-to-day management of the organization.

Organizations apply for reaccredidation on a yearly basis.

According to TNO, QMIC "sets a low ceiling for quality standards that is balanced by robust feedback mechanisms that can access the provider as well as the TIP's databases," that is, although the quality standards themselves might not be too onerous for information providers, nonetheless, the standards will be validated through strong feedback mechanisms by citizens and third-party certifying companies.

**Sustainability issues**

See under "URAC," above.

### HON Code [24- 25]

**Launch Date**

1996.

**Responsible Organization**

Health on the Net (HON) Foundation in Geneva Switzerland [26].

The HON Code is probably the earliest quality initiative on the health Internet. The HON Code logo can be found on more than 3000 health-related websites. Nevertheless, despite the profound changes taking place in the health Internet sector, the HON Code has not been updated since its creation.

The Foundation is a not-for-profit organization established in 1995, funded primarily by the State of Geneva and the Geneva Ministry of Health. HON receives additional support and donation and grant money from a variety of sources, including the Swiss Institute for Bioinformatics and Sun Microsystems.

More recently, the Foundation is seeking formal recognition by the United Nations as a Non-Governmental Organization.

**Intended target users**

Health information providers, consumers, and medical practitioners.

**Objectives**

Guide laypersons and medical practitioners to useful and reliable online medical and health information.

**Approach**

Self-regulatory quality seal displayed on sites that conform to the HON Code.

This is the approach definition used by HON: "The HONcode is not an award system, nor does it intend to rate the quality of the information provided by a Web site. It only defines a set of rules to:

- Hold Web site developers to basic ethical standards in the presentation of information;
- Help make sure readers always know the source and the purpose of the data they are reading

**Process**

The HON Code was developed as an internal process in consultation with Webmasters, information providers, patients, and citizens.

**Implementation mechanisms**

The HON Code sets 8 principles for basic ethical standards for the health Internet. Sites that conform to those 8 principles are allowed to display the active HON Code logo on their pages.

This is a self-certification system that has little control of how the logo is used. However, HON does try to police the use and abuse of its logo through the following mechanisms:

- An alert of breach is sent to the provider
- A warning is issued to the offending site
- Removal of the live link between the HON logo on the provider site and the HON site

HON also provides an online checklist questionnaire (Site-Checker) that can help consumers assess whether a given site conforms to the HON Code principles.

**Sustainability issues**

The HON Code places a burden on citizens through the need by those citizens to verify for themselves what is essentially a claim by the information provider. It is vulnerable to availability of funding for HON Foundation, which will be required to maintain the currency of the Code.

### European Commission: Quality Criteria for Health Related Websites [27]

**Launch Date**

June 2001.

**Responsible Organization**

European Commission

DG Information Society: Information Society Technologies: Systems and Services for the Citizen

DG Health and Consumer Protection: Public Health

**Intended target users**

European Union member states.

**Objectives**

Produce a European Commission Communication on Good Practice Guidelines for the Health Internet. The scope of this Communication will be health-related information society services, and covers health information and services on the Internet. This scope does not extend to the category of products.

A European Commission Communication differs from a Directive in that it has no binding power on the member states to incorporate into domestic law. It is issued for guidance and to recommend a particular course of action. A Communication, however, can be used in legal arguments and a judge may cite it in cases of non-compliance.

**Approach**

EC "soft power" based on consensus building and guidance to member states on a voluntary code of conduct based on quality criteria.

**Process**

- Expert, stakeholder and EC civil servants workshop
- Drafting Group
- Online discussion
- Public consultation

**Implementation mechanisms**

Non-binding EC Communication guidance to member states.

Sustainability issues:

- Acceptance and implementation by member states will determine usefulness
- Like codes of conduct, it places a burden on citizens

### OMNI [28- 29]

OMNI, Organizing Medical Networked Information, is part of the BIOME gateway hub.

**Launch Date**

1996.

**Responsible Organization**

UK (United Kingdom) Joint Information Services Committee (JISC), which also funds the program.

**Intended target users**

OMNI targets medical students, researchers, academics, and practitioners. OMNI is currently widening its appeal to consumers and is developing a set of quality-evaluation criteria for complementary and alternative medicine.

**Objectives**

Provide access to evaluated, quality Internet resources in the health and life sciences, aimed at students, researchers, academics, and practitioners.

**Approach**

Expert third party evaluation of networked medical information based on the OMNI "Evaluation Guidelines" created by the OMNI "Advisory Group on Evaluation Criteria."

**Process**

- OMNI Evaluation Guidelines created by the OMNI "Advisory Group on Evaluation Criteria
- Description and cataloging of resources based on the BIOME "Cataloguing Guidelines"
- Collection development policy

**Implementation mechanisms**

OMNI uses a standard web interface to search the catalogs of reviewed resources. It has catalogued approximately 4000 sites to date.

**Sustainability issues**

The OMNI team faces a Herculean task in keeping up with new sites, products, and services that are emerging all the time, let alone keeping the original evaluation up to date.

This places a burden on the team in terms of human and financial resources and at the same time, the OMNI program places a burden on citizens in terms of their need to understand and assess the quality criteria applied to the catalogs.

### DISCERN [30- 31]

**Launch Date**

1999.

**Responsible Organization**

The DISCERN Project Team based at the Division of Public Health and Primary Care at the Institute of Health Sciences of the University of Oxford England. DISCERN is funded by UK National Health Service Executive Research and Development Programme.

Intended target users:

- Citizens seeking information on treatment choices for certain conditions
- Authors and publishers of information on treatment choices

**Objectives**

- Enable consumers to judge the quality of written information on treatment choices
- Facilitate the production of high quality evidence-based patient information

**Approach**

Citizen evaluation of Web sites carrying treatment information based on an aggregated assessment derived from a predefined questionnaire (the instrument).

**Process**

- Expert panel analysis. Panel composition: clinical specialists, self-help group representatives, general practitioners, consumer health information expert, lay medical publisher, health journalist, health consumer representative, Community Health Council representative, Plain English Campaign representative, and NHS Centre for Reviews and Dissemination representative
- Development of draft instrument
- Instrument testing
- Selected stakeholder testing
- National pilot
- Development of a standardized quality index derived from the questionnaire

**Implementation mechanisms**

Subjective rating system for decisions on treatment choices based on the questionnaire.

**Sustainability issues**

DISCERN places a burden on the citizens, as they would have to (a) understand the quality criteria behind the questionnaire, (b) have the commitment to fill in the questionnaire, and(c) have the ability to understand the meaning of the score value.

### Guidelines for Medical and Health Information Sites on the Internet - Principles Governing AMA Web Sites [32- 33]

**Launch Date**

2000.

**Responsible Organization**

American Medical Association (AMA)

**Intended target users**

Web sites of the American Medical Association, Medem (http://www.medem.com) and other providers and users of medical information on the Web.

**Objectives**

Govern the Web sites of the AMA, AMA Publications, and Medem.

**Approach**

Rules of conduct that govern health information on the Web are based on those that govern medical journals, including rules of peer review, authorship, full disclosure of funding and sponsorship, editorial independence, separation of content and advertising, and the principles of privacy and confidentiality based on the principle of informed consent.

**Process**

The development of these guidelines began in 1999. An AMA staff committee, composed of the listed authors, was organized to review the existing individual guidelines and draft a single document that would provide principles to govern the presentation and functionality of the 4 major areas for which quality standards were needed: content, advertising and sponsorship, privacy and confidentiality, and e-commerce.

Committee members reviewed initial drafts and consensus was reached on the content of each of the 4 principles. The document was then reviewed internally and externally by experts in ethics, publishing, government regulations, law, and medical informatics, and by the AMA Online Oversight Panel. After subsequent revision, the document was reviewed by the Executive Committee of the AMA Board of Trustees and was approved on February 28, 2000. The guidelines underwent peer review and were published in JAMA on March 22, 2000.

**Implementation mechanisms**

These are governance tools intended for use by the developers of the AMA's Web sites and of the Medem Web site. Other organizations have adopted these guidelines or used them as the basis for their guidelines. The AMA does not ensure compliance with the guidelines for organizations other than itself and Medem.

### Quality Standards for Medical Publishing on the Web [34]

**Launch Date**

1996.

**Responsible Organization**

British Healthcare Internet Association (BHIA).

This is a not-for-profit organization whose mission is better health care through the application of Internet technologies. It is funded by membership fees. The membership is open to anyone who supports the mission of the organization, and currently comprises clinicians, publishers, Web site developers, information providers, information technology professionals, health care managers, government officers, and academics. The BHIA has 120 members. The organization is currently not active.

**Intended target users**

Medical Webmasters and medical information providers.

**Objectives**

Better quality of medical information on the web.

**Approach**

A set of quality criteria for improving the quality of on-line medical information focusing on the content of Web sites.

**Process**

Paul Galloway authored the draft of the quality standards. That draft was submitted to the membership for comments and amendments. The final document was approved by the membership as a BHIA Recommendation in an online consensus process.

**Implementation mechanisms**

Guidance to medical Webmasters.

There has been no further development of the criteria and it is not known if they are used in practice and, if they are used, it is not known how they are used.

### The Health Summit Working Group (HSWG) Criteria for Assessing the Quality of Health Information on the Internet: IQ Tool [35]

**Launch Date**

1997/1998.

Originally funded by Mitretek Systems Inc [36], the HSWG IQ Tool is one of the earliest tools-based scoring methods for assessing the quality of health websites.

Mitretek Systems Inc, although it morally supports the use of the tool, no longer funds the project. The project is no longer developed or maintained by any organization.

The tool was developed using an expert-group consensus process. The work resulted in a set of criteria that have a weighted scoring system.

Users deploy the tool when visiting a Web site they wish to evaluate and have to go through the process of completing the questionnaire in order to arrive at a quality score.

It is not know whether this tool is in use.

### The International Federation of Pharmaceutical Manufacturers Associations (IFPMA) Code of Marketing [37]

**Launch Date**

1981/1982.

Last major revision: 1994.

The IFPMA Code of Marketing sets out universal principles for ethical marketing conduct for use in countries where a more-demanding national code of conduct does not exist.

The Code applies to ethical pharmaceutical products and stresses the need to respect local and national laws; however, its scope also includes the controversial issue of direct-to-consumer marketing of ethical pharmaceutical products.

The IFPMA Code does not have specific clauses on Internet health information; however, it includes the addendum below addressing the issue of the Internet in a vague and general way:

Addendum 1:

Use of the Internet

The research based pharmaceutical industry, represented by the IFPMA, strongly supports the right to use the Internet as a means of providing accurate and scientifically reliable information on medicines in a responsible manner, for the benefit of patients, healthcare professionals and other appropriate parties. Recognizing patient safety is of paramount importance, IFPMA's goal is to encourage the appropriate use of the Internet.

The IFPMA considers that there should be open access to all information put on the Internet by pharmaceutical companies. It accepts that there are national differences in the laws and regulations governing the promotion of medicines.

Many pharmaceutical companies have established corporate sites on the Internet, which provide information about the company. Non-product related information is outside the scope of the IFPMA Code.

The IFPMA recognizes that certain uses of the Internet may fall within the scope of the IFPMA Code of Pharmaceutical Marketing Practices. The following points concern product-related information:

The identity of the pharmaceutical company and of the intended audience should be readily apparent. The content should be appropriate for the intended audience. Links should be appropriate and apparent to the intended audience. Country-specific information must comply with local requirements.

The IFPMA Marketing Code, does not specify in detail what aspects of the Code apply to health information (on, for example, diseases and conditions) attached to products or produced and published by pharmaceutical companies on the Internet.

It would be interesting to determine the level of acceptance and implementation by member pharmaceutical companies. It is important to determine how this guidance from the IFPMA differs from any criteria set by pharmaceutical companies for their own internal processes in general.

### Other pharmaceutical organizations that may have an impact on quality of Internet health information

Listed below are some of the organizations that may have an impact on quality of Internet health information of pharmaceutical companies in the relevant jurisdictions. Almost all of these organizations will have some sort of reference to quality standards of health information published by their constituencies on the Internet. The list below was adapted with permission from the InPharm Web site http://www.inpharm.com/db/ieindex.html.

All Web sites in this list: [accessed 2001 Oct 4].

- General Pharmaceutical Inspectorate (Belgium) URL: http://www.afigp.fgov.be/
- Medicines Evaluation Board (Netherlands) URL: http://www.cbg-meb.nl/uk/overcbg/index.htm
- European Agency for the Evaluation of Medicinal Products URL: http://www.emea.eu.int/
- European Department for the Quality of Medicines-European Pharmacopoeia on the Web URL: http://www.pheur.org/
- European Society of Regulatory Affairs URL: http://www.esra.org/Resource.phx/community/mainpage/mainpage.htx
- US Food and Drug Administration (FDA)-Center for Drug Evaluation and Research URL: http://www.fda.gov/cder/
- US Food and Drug Administration URL: http://www.fda.gov
- IDRAC, International Drug Registration URL: http://www.eu.imshealth.com/idrac/
- International Conference on Harmonisation of Technical Requirements for Registration of Pharmaceuticals for Human Use URL: http://www.ifpma.org/ich1.html
- Irish Medicines Board URL: http://www.imb.ie
- UK Medicines Control Agency URL: http://www.mca.gov.uk/
- UK National Institute for Biological Standards and Control URL: http://www.nibsc.ac.uk/
- Pharmaceutical and Medical Devices Evaluation Center (Japan) URL: http://www.nihs.go.jp/pmdec/outline.htm
- Prescription Pricing Authority (UK) URL: http://www.ppa.org.uk/
- Regulatory Affairs Professionals Society (RAPS) URL: http://www.raps.org/
- Medical Products Agency (Sweden) URL: http://www3.mpa.se/ie_engindex.html
- The United States Pharmacopoeial Convention Inc URL: http://www.usp.org/

## Discussion

The discussion is organized into:

- Key mechanisms of the health information quality initiatives
- **Sustainability issues**
- Enforcement issues
- Adequacy of approach and enforcement provisions
- Scope and reach
- Gaps that need to be addressed

### Key mechanisms of the health information quality initiatives

The starting block of all the initiatives is a set of quality criteria. These sets of criteria range from the simple common-sense perspective of Paul Galloway and the peer-review journal approach of William Silberg et al [38], to the extensive and elaborate sets of quality criteria of URAC and MedCERTAIN.

All the sets of criteria derive from very similar roots and differ only in the language and expression of those roots. Briefly, these roots are the principles of honesty, privacy, confidentiality, accuracy, currency, provenance, consent, disclosure, and accountability.

The initiatives' developers chose different mechanisms to transform these sets of quality criteria into programs of Internet health information governance. On the surface, these key mechanisms seem to be many; however, a closer look reveals that these key mechanisms (or philosophies) belong to one of 3 underlying mechanisms.

These 3 key mechanisms can be summarized as follows:

#### Codes of conduct or ethics

These are based on principles of ethical behavior and sets of quality criteria. Almost all the quality criteria used by the initiatives converge at some point or another. It is only the language used to describe the criteria that is different. For example, the ehealth Code of Ethics states: "Disclose information that if known by consumers would likely affect consumers' understanding or use of the site or purchase or use of a product or service" in the "Candor and honesty," section, whereas we find Hi-Ethics Inc states: "We will inform consumers who use our Internet health services of the risks, responsibilities, and reasonable expectations associated with their use of our services" in the "Transparency of Interactions, Candor and Trustworthiness" section.

Codes of conduct rely on self-certification by participating Web sites, for example, those displaying the HON Code. This self-certification process is nothing more than a claim or a pledge that has little enforceability.

#### Third-party certification

This requires recurrent validation of compliance with a set of standards. These standards may or may not be based on some of the codes of conduct and ethics discussed here. In all cases, third-party certification requires payment of fees to the certifying company.

Special note on the metadata element of the MedCERTAIN initiative:

- The MedCERTAIN initiative is essentially a third-party certification program. However, the developers describe the program as third-party description and annotation rather than certification. Thus, it would seem that the MedCERTAIN process contains both elements of "self-description" and third-party evaluation.
- The program uses sets of detailed quality tags (quality vocabulary) embedded in the technical infrastructure of documents to describe the content on offer (similar in concept to food labels) [39].
- Users of health information would be able to filter the content they receive or view based on, initially, being aware of the metadata sheet, and later, automatically via the setting of personal preferences within the browser environment (downstream filtering).
- Third parties endorsing, describing, or evaluating other sites, for example, gateways and libraries (such as MedlinePlus or OMNI); professional associations; or certifying organizations (such as URAC), can use this metadata language to describe information content or information providers.

#### Tool-based evaluation

This is mostly based on a predefined questionnaire that would yield a certain "quality score" for the content under evaluation.

Tool-based evaluation is primarily intended for use by citizens, who would invoke the particular tool to assess the quality of a given site. This process differs from self-certification and logo-bearing sites, for example, under the HON or MedCERTAIN programs [Comment added by the editor: However, note that MedCERTAIN enables the development of intelligent "next generation" tools aggregating and interpreting metadata, see editorial]

Advantages and disadvantages of the three key mechanisms are listed in Table 4.

#### Sustainability issues

The following unresolved issues cast doubts over the ability of the various quality initiatives to survive what is largely an unregulated and often anarchic medium.

#### Burdens

All the quality initiatives under discussion place a number of burdens on producers and users of health information and on others. These burdens are seen as a serious threat to the sustainability and maintenance of the quality standards. The burdens fall on one or more of the following:

- Citizens: by having to care about, bother about, understand, and apply the methodology of any given initiative
- Providers of health information: by having to understand, interpret, and specify the program, and apply the program to their operations
- Third-party accreditation organizations: by having to acquire sufficient knowledge and a customer base to make their business viable
- Organizations that sponsor initiatives: by having to develop and maintain their programs, often under very adverse financial conditions, for example, many of these organizations rely on donations and grant money that might not always be forthcoming
- Clinicians and other health care workers: by having to care about, bother about, understand, and apply the methodology of any given initiative

#### Currency and maintenance

The ability of the organizations sponsoring the quality initiatives to maintain their initiatives up to date can be vulnerable to scarcity of funding (for voluntary and non-profit organizations and for-profit and fee-based organizations alike), and to low acceptance of quality programs. This is particularly acute at times of rapid change in the health Internet or market downturns that affect donor contribution or membership gains adversely.

#### Funding

Most of the initiatives rely on donation and grant money to maintain and develop their work. This makes them vulnerable to conditions outside the their control at best, and to potential undue influence at worst.

**Table 4:** Advantages and Disadvantages of the 3 Key Mechanisms of the Health Information Quality Initiatives

**Mechanism**	Advantages	Disadvantages
**Codes of conduct**	Usually developed by broad-based participation Create useful stakeholder consensus Amenable to sector-specific interpretation Can be updated as necessary with relative ease Can be implemented by any organization, large or small Create synergy between corporate objectives and ethical environments	Implementation is by a nonbinding pledge Open to abuse Potential for misinterpretation of principles Require non-specific organizational change that is difficult to measure Difficult to measure utilization by Web sites and citizens Difficult to measure effectiveness
**Third-party certification**	Provides independent validation and revalidation Can be objective Forces organizational change in terms of ethical culture, audit, and accountability Forces provider education Clear criteria that are consistently applied; but, criteria can be quite clear, without being well-considered and defendable Relatively easy to measure utilization (in the case of fee-for-service programs) Can be used as a quality differentiator	High cost to providers Exclusion of smaller and other deserving providers due to cost or required organizational change User indifference (don't know, don't care) Provider ambivalence (don't care, can't do) Enforcement relies on withdrawal of accreditation; this has a weak impact Labor and resource intensive in the case of manual review and certification (eg, MedCERTAIN and OMNI)
**Tool-based evaluation**	Consistency of process	Semblance of objectivity User indifference Difficult to measure utilization by Web sites and citizens Difficult to measure effectiveness Narrow "expert"-only participation in developing the questionnaires that underlie the tool False sense of security Difficult to maintain currency Difficult to establish validity

#### Cost

The initiatives most likely to command credibility are those in which there is independent third-party certification. Yet, third-party certification places a financial burden on the organization seeking certification, because of the high level of fees charged for accreditation or certification as well as the cost of the required organizational change. There are no credible means of offsetting that cost or ameliorating it for small yet useful providers.

#### Acceptance

Most of the initiatives rely on establishing a critical mass of acceptance. It remains difficult to assess the degree of current or future acceptance for any given initiative.

#### Market conditions

Prevailing market conditions play a crucial role in determining corporate policies. There is a real fear that market downturns would affect quality implementation adversely. Equally, in times of plenty, corporate sights might be set on other things. In market downturns, quality and ethics might be victims of cutbacks. In times of plenty, quality and ethics might be relegated behind profit making, unless they are seen to be a competitive advantage.

#### User indifference or ambivalence

Users need to care about, bother about, understand, and implement the requirements of the quality initiatives; but, users

- might not be aware of the issues of quality or the existence of quality initiatives and programs
- might care about and be aware of the quality initiatives, but not understand the initiatives and the requirements these initiatives place on them
- might not care too much about quality issues
- might be aware of and care about quality of health information, yet not bother too much about following what is required of them by the various initiatives

#### Enforcement issues

All except one of the quality initiatives discussed here are based on self-regulation systems that are applied voluntarily and which have enforcement mechanisms that rely on the unconvincing notions of self-declaration, self-certification, or withdrawal of accreditation. The exception is the initiative by the American Medical Association's program, which is enforced by the AMA corporate body.

Even when third-party certification and revalidation are the requirements, it seems that the only enforcement sanction is withdrawal of accreditation, which might not be a very effective enforcement sanction or might have a weak impact on the Web site in question.

Enforcement provisions of the voluntary initiatives discussed here do not seem to be adequate in view of the lack of credible sanctions for non-compliance and worthy rewards for compliance.

There are so many disparate constituencies within the domain of Internet health information that it is quite difficult to see how sector-specific self-regulation can be successful without further and deeper characterization, interpretation, and specification to identify the needs of specific sectors of information providers.

Enforcement mechanisms used are summarized as follows:

- None
- Self-certification or a pledge, without validation
- Third-party withdrawal of accreditation and revalidation by a third party

#### Adequacy of approach and enforcement provisions

The following inadequacies in the approach and enforcement provisions have been identified.

The potential high cost of implementing quality standards, particularly by small and voluntarily-funded entities.

The inability to formulate mechanisms that address the quality of the "pseudo-health" sector:

- The existence of the pseudo-health segment of information producers and users complicates efforts to introduce quality standards for health information on the Internet. This is the "gray market" of health information and it includes practices and remedies that have not yet been proven empirically or anecdotally; some unproven wellness products; misleading nutrition information; dubious mineral, plant and animal alternatives to pharmaceuticals; and the dissemination of untypical personal experiences.
- This pseudo-health sector presents the most challenges in ensuring the dissemination of good quality health information and practices. Whereas reputable producers of health information on the Internet would not have many problems complying with most of the quality criteria under discussion, the pseudo-health sector will probably remain outside the philosophy of applying quality standards in a self-regulatory manner. This is because much of the motivation for this sector is mainly financial gain through fraud and deception.
- It is important to distinguish this sector from the alternative and complementary health-care sector. Many of the disciplines in the latter sector have an important and legitimate role to play in the health and well-being of many people.

The lack of credible incentive and deterrence in implementing quality policies by information providers.

The size of the burden placed on the various players.

The absence of strategies for auditing utilization of the initiative's implementation.

The absence of adequate sector characterization and specification.

Lack of clarity of the language and terminology of quality [40].

Absence of any clear mechanisms for cooperating with regulatory authorities to implement programs of co-regulation.

Absence of clear strategies to extend the proposed protection measures on a global scale.

Inadequate response to liberal and conservative arguments against development of standards for health information on the Internet.

#### Scope and reach

Most of the initiatives target citizens as the ultimate beneficiaries without recognizing the scale and practical challenges of citizen-education issues and the diverse levels of critical appraisal skills among citizens.

That focus on citizens ignores the other participants in the matrix of health information: for example, clinical services providers, research communities, public health institutions, and policy makers worldwide. It also neglects the crucial role of doctors and other health-care workers as effective arbiters of quality of health information.

Many of the initiatives do not have a universal reach, for example, Hi-Ethics Principles and the AMA Guidelines.

All the initiatives stem from a Western orthodox view of health and health information. This is particularly noticeable whenever evidence is mentioned. Examination of quality initiatives originating from non-English speaking organizations might provide a different view.

None of the initiatives address the issues and needs of communities that are still catching up with, deprived of, or oblivious of the information revolution because of poverty, lack of access to content and connectivity, or the capacity to produce and disseminate health information.

With the exception of the eHealth Code of Ethics, MedCERTAIN, and the HON Code, the initiatives are published in the English language only. Not only does that limit the benefits accrued to non-English speaking citizens, but it also prohibits non-English speakers from contributing to the formulation of Internet health information standards.

#### Gaps that need to be addressed

Many of the gaps that need to be addressed in future quality initiatives are discussed above. In summary, these gaps are (in order of priority):

- Enforcement provision
- Burdens
- Sustainability
- Scope
- Reach
- Definition of quality
- Language and terminology
- Meaningful dialogue with regulatory authorities and entities outside the health Internet sphere
- Strategic and operational programs of co-regulation
- Audience characterization and specification
- Provider education
- Language and readability barriers
- Audit strategies for quality-program utilization
- The pseudo-health sector
- The needs of clinicians and other health-care workers
- The role of clinicians and other health care workers as effective intermediaries of health information quality

### Conclusions

The complexity of the issues surrounding quality of health information in the context of the health Internet has been shown.

Some of the key self-regulation initiatives of Internet health information quality have been described and analyzed. The various initiatives have been compared in a number of ways.

The "Discussion" section, above, clarifies and discusses the issues and requirements for the further development of Internet health information quality.

#### Conclusion 1

Internet health quality initiatives discussed here have 1 of 3 mechanisms. It seems that 1 or more of 3 mechanisms would underpin future development of quality initiatives. These key mechanisms are:

- Codes of conduct or ethics
- Third-party certification of compliance (accreditation)
- Tool-based evaluation of quality

#### Conclusion 2

Based on the analysis of quality initiatives and the discussion above, it is proposed that a successful quality program has these 3 essential elements:

- A set of health information quality criteria
- An educated, interested, and active citizen
- Credible enforcement instrument(s)

None of the initiatives discussed in this paper comprise all 3 elements convincingly.

These 3 elements must be taken into account in any future developments and implementation of health information standards.

#### Conclusion 3

The current batch of quality initiatives for Internet health information reveals many gaps that need to be addressed. These gaps are discussed and listed in this paper. The most serious of these gaps are the excessive burdens placed upon citizens and the cost of implementing credible programs providing accreditation and enforcement.

Further examination and the addressing of these gaps is essential for any future development work on Internet health information governance.

#### Conclusion 4

More research is needed to further clarify the complexities of Internet health information. Of special interest are the governance mechanisms that need to address quality of information content and information value, the context and relevance of the content of information, the educated interested citizen, and the desired instruments that would strengthen any envisaged enforcement provision.

#### Conclusion 5

There is an urgent requirement to examine the needs of the developing world and the info-poor in relation to quality of Internet health information, products, and services. This is a reflection on how poorly the current batch of quality initiatives have addressed those needs.

This examination would include determining whether or how quality standards can help developing countries, especially where regulatory agencies are weak or nonexistent; or where there is excessive, uninformed, or onerous regulation.

#### Conclusion 6

There are no current mechanisms for ensuring the quality of Internet health information in relation to the pseudo-health sector. This sector will probably remain outside the efforts to implement governance of health information quality.

#### Conclusion 7

The quality initiatives discussed here have not addressed the thorny issue of alternative and complementary disciplines outside the orthodox view of health care.

These disciplines differ from those of the pseudo-health sector in that they have a legitimate place in health care, whereas the pseudo-health sector is essentially about fraud and quackery.

#### Conclusion 8

Language, whether in tongue or in syntax, remains a major obstacle to the dissemination of good practices and the education of citizens and information providers alike.

#### Conclusion 9

There is a need for coordination and harmonization of the efforts striving towards quality health information on the Internet. This extends to the key players in both the self-regulation and the mainstream and regulatory camps, and includes regional and international bodies, the health care products industry, foundations with an explicit interest in Internet health information, private and corporate interests, and citizen and country representation and participation.

#### Conclusion 10

There are concerns and criticisms directed against establishing models of governance for Internet health information quality. These arguments come from different perspectives and take different routes but arrive at the same destination. These concerns include:

- Users are ambivalent or indifferent about quality through ignorance, lack of caring, or low priority
- Quality programs that are not rigorously enforced and validated might produce a false sense of security
- Traditional media did not require quality standards; therefore neither should the new media
- Brand loyalty is more important than quality seals; the Internet has no center; therefore, it does not need central control; and, kitemarking (referring to the application of a kite-shaped mark granted for use on goods approved for use by the British Standards Institution) the Internet is like "kitemarking the west wind" [41].
- Freedom of speech
- Free market forces
- The enormous practical and logistical difficulties associated with implementing quality programs are a barrier to implementation

As arguments, they are in no way compelling or well thought out. Indeed, they seem more to be descriptions of behavior for which no rationale for taking them seriously is given by those who invoke them.

It is concluded that these arguments should be countered with a coherent strategy for health information quality governance that can unite the stakeholders in an effort to reduce the risk of harm to citizens throughout the world (see "Recommendations," below).

### Recommendations

In any new field of human endeavor there emerges at the beginning a group of individual pioneers, visionaries, and entrepreneurs. These individuals, by their nature, kick-start the standards setting process for that new field. They tend to do this either as individuals or through forming into associations. These are mostly voluntary organizations that rely on the enthusiasm and energy of their members, and often struggle to meet the financial and management demands that are placed upon them.

The new field of and the early work in standards development eventually attract the attention of society's mainstream players or the gap between the pioneers and the mainstream players narrows enough for the mantle to pass onto the mainstream players.

The health Internet has been no different. We are probably at the cusp of that convergence to the extent that it has become imperative to bring together the 2 camps of active pioneers and mainstream players in a coherent and coordinated process to develop the next generation of quality standards.

### The need for global leadership

Quality of Internet health information is important, because it has the potential to benefit or harm a large number of people. It has this potential because of the nature of the Internet and the Internet's rapid worldwide spread.

The quality of Internet health information is too important to be left to the anarchy of the Internet or the vagaries of the free market, or to be conducted in a haphazard uncoordinated way.

The absence of clear, credible, and trusted leadership in the sphere of Internet health information compounds the problems of quality and trust relationships among people who use the Internet for health purposes.

The author believes that there is a need for clear leadership on a global scale to achieve the yet-unfulfilled promise of information and communication technologies of better health for all.

This global leadership needs to take the following steps to assume that leadership role:

- Bring together the key players of both the pioneer and mainstream camps in a coherent effort that can benefit all citizens of the world
- Harmonize a global framework for Internet health information quality standards
- Act as intellectual and technical knowledge resource for the world
- Provide custody and good stewardship of the evolving standards
- Implement a program to ensure the prevention of harm to communities and nations yet to be exposed to powerful free-market-economy forces
- Safeguard the interests of the info-poor
- Provide impartial advice and guidance not constrained by politics or geography
- Facilitate the dialogue between the interested parties of self-regulation and the regulatory authorities towards the creation of programs of co-regulation
- Work towards "the global public good" and the benefit of all citizens of the world

### The role of the World Health Organization (WHO)

In line with the WHO's global role in setting norms and standards and assisting member states to implement these norms and standards, the organization has a crucial role to play in developing norms and standards for Internet health information quality.

We recommend that the WHO's activity in this sphere should include the following terms of reference:

- The fulfillment of 3 crucial requirements:
  o Increase the understanding of Internet health information quality standards
  o Assess the impact of implementation of such standards at country level
  o Recommend a framework of action for Internet health information quality
- Bringing together key players from the stakeholder communities in a coherent and coordinated manner.
- Ensuring good stewardship of ethics and quality standards development.
- Consensus building among the various interested parties.
- Facilitating the steering of these programs towards the establishment of universally-agreed quality standards for Internet health information.
- Providing a world resource for Internet health information quality thinking and research.
- Coordinating educational and training activities relating to quality.
- Disseminating good practices throughout the world and assisting member states in the implementation of those good practices.
- Working with the private sector to help advance the cause of quality of Internet health information.

## Abbreviations

AMA: American Medical Association
BHIA: British Healthcare Internet Association
EC: European Community
EU: European Union
HIDDEL: Health Information Disclosure, Description and Evaluation Language
HON: Health on the Net
HSWG: The Health Summit Working Group
HSWG IQ Tool: The Health Summit Working Group-Criteria for Assessing the Quality of Health Information on the Internet: IQ Tool
IFPMA: International Federation of Pharmaceutical Manufacturers Associations
ISO: International Organization for Standardization
IT: Information Technology
NBF: Notified Body Function
OMNI: Organizing Medical Networked Information
QMIC: TNO Quality Medical Information and Communication
TIP: Trusted Independent Party
UK: United Kingdom
US: United States
WHO: World Health Organization
XML: Extensible Markup Language
